# Quaternary functionalized mesoporous adsorbents for ultra-high kinetics of CO_2_ capture from air

**DOI:** 10.1038/s41598-020-77477-1

**Published:** 2020-12-08

**Authors:** Tao Wang, Xinru Wang, Chenglong Hou, Jun Liu

**Affiliations:** 1 State Key Laboratory of Clean Energy Utilization, College of Energy Engineering, Zhejiang University, Hangzhou, 310027 People’s Republic of China; 2 School of Electric Power, North China University of Water Resources and Electric Power, Zhengzhou, 450045 People’s Republic of China

**Keywords:** Carbon capture and storage, Materials for energy and catalysis

## Abstract

Obstacles to widespread deployments of direct air capture of CO_2_ (DAC) lie in high material and energy costs. By grafting quaternary ammonium (QA) functional group to mesoporous polymers with high surface area, a unique DAC adsorbent with moisture swing adsorption (MSA) ability and ultra-high kinetics was developed in this work. Functionalization is designed for efficient delivery of QA group through mesopores to active substitution sites. This achieved ultra-high kinetics adsorbent with half time of 2.9 min under atmospheric environment, is the highest kinetics value reported among DAC adsorbents. A cyclic adsorption capacity of 0.26 mmol g^−1^ is obtained during MSA process. Through adsorption thermodynamics, it is revealed that adsorbent with uniform cylindrical pore structure has higher functional group efficiency and CO_2_ capacity. Pore structure can also tune the MSA ability of adsorbent through capillary condensation of water inside its mesopores. The successful functionalization of mesoporous polymers with superb CO_2_ adsorption kinetics opens the door to facilitate DAC adsorbents for large-scale carbon capture deployments.

## Introduction

Limiting global warming to less than 1.5 °C over preindustrial levels with limited or no overshoot projects the Negative Emission Technologies (NETs)^1^, which is expected to remove CO_2_ on the order of 100 Gt by midcentury^2^. As an important component of NETs portfolio, direct air capture (DAC) of CO_2_ by chemicals has advantages of low environmental risk, convenient feedstock for CO_2_ utilization and unlimited capacity in reducing atmospheric CO_2_ level^3,4^. However, NETs are always challenged by low kinetics issues under the ultra-low CO_2_ partial pressure of 40 Pa in air. Under this atmospheric environment, the rates of CO_2_ uptake by photosynthesis and ocean absorption are on the order of 10^–6^ and 10^–8^ mol m^−2^ s^−1^, respectively^5,6^.

Kinetics of artificial material of DAC can be hundreds of times higher than that of natural process. Decades of sorbents development for flue gas capture provides various functional parts referenced to DAC, ranging from alkali hydroxides to solid amines^4,7^. As both functionalization and CO_2_ adsorption need channels for molecule diffusion, porous DAC adsorbents have aroused extensive concerns. During functional group impregnation, researchers found that amine molecules are prone to first filling small pores (< 10 nm) due to their relatively high surface potential^8,9^. This would result in complete filling of pore channels and limiting CO_2_ diffusion after functionalization, especially for adsorbents with microporous. To avoid this outcome, low functional group loading (e.g. < 9 mmol N g^−1^) is suggested to keep a certain amount of mesopores (e.g. 1 cm^3^ g^−1^ for aerogel^10^, 0.35 cm^3^ g^−1^ for mesoporous alumina^11^). In situ polymerization with amine-containing monomers, e.g., linear poly-l-alanine^8^ or hyperbranched aminosilica^12^, is also a preference for keeping the mesoporous structure of adsorbents.

Of particular interest to this work is chemically grafting quaternary ammonium (QA) groups on mesoporous material for DAC adsorption. The QA groups possess the ability of moisture swing adsorption (MSA) which employs water to trigger the desorption, rather than heat or electrical energy. Therefore, the MSA could have a lower energy consumption than temperature-swing adsorption (TSA) process^13^. The pore structures are introduced to MSA adsorbent in this work to enhance its molecular diffusivity. The grafting of QA cations with strong ionic bonding towards anions can avoid the overlap of functional groups inside mesopores^14^. Also, the mesopores can provide more surface area for functionalization, compared to macroporous supports^4,7,15,16^ or cellulose fiber with limited pore structure^17^. This further provides sufficient channels for CO_2_ diffusion and high efficiency ammonium sites for CO_2_ to be captured. On the other hand, moisture swing adsorption (MSA) is known as water can distinctly alter the binding energy of QA ion pair to CO_2_ through the Brønsted base mechanism^15,18^. Thus, water behavior in mesopores, such as diffusion and capillary condensation, will ultimately project on the performance of moisture swing adsorbents.

## Results

### Design and fabrication of QMPRs

**Figure 1:**
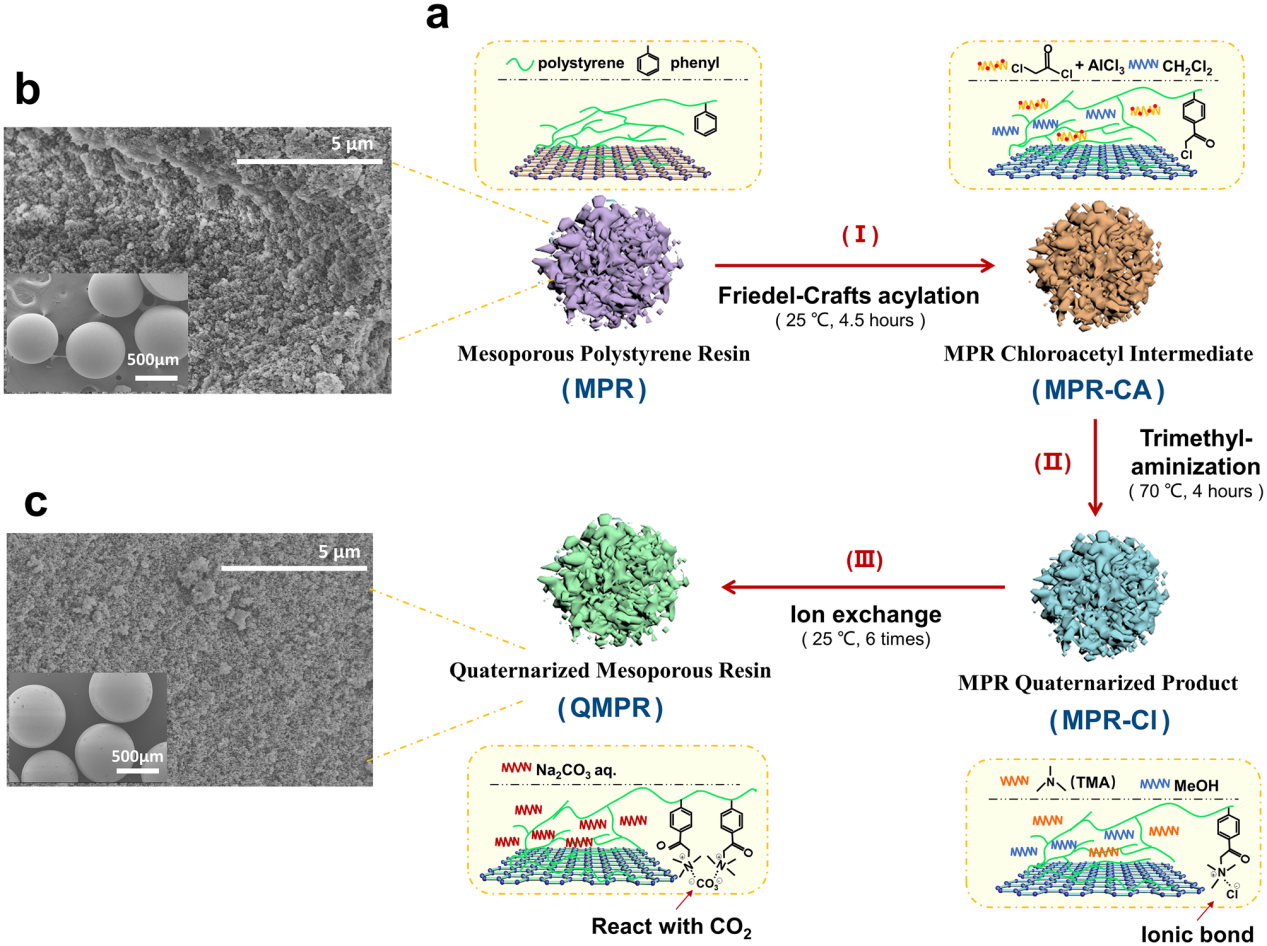
Fabrication and morphology of QMPRs. (**a**) Schematic illustration of QMPR adsorbents preparation through three-step process. (**b**,**c**) SEM images of the MPRs and QMPRs (**b** represents MPR-2, and **c** represents QMPR-2) including both particle and internal pore morphology.

Quaternary ammonium functionalized mesoporous adsorbents (QMPRs) are produced though a three-step process illustrated in Fig. 1a. Using this approach, three types of porous QMPRs based on MPRs with similar particle size (500–600 µm as shown in Fig. 1b,c) have been prepared (FTIR analysis and surface morphology are shown in Supplementary Fig. 1). During synthesis process, dichloromethane and methanol were selected as swelling agents, as they can overcome steric hindrance and permeate reagents into active substitution sites at a sufficiently swelled state^19^. Carbonate is selected as the counter anion to QA group to build a strong interaction with CO_2_ in air^15^.

### Characterization of pore structure

**Figure 2:**
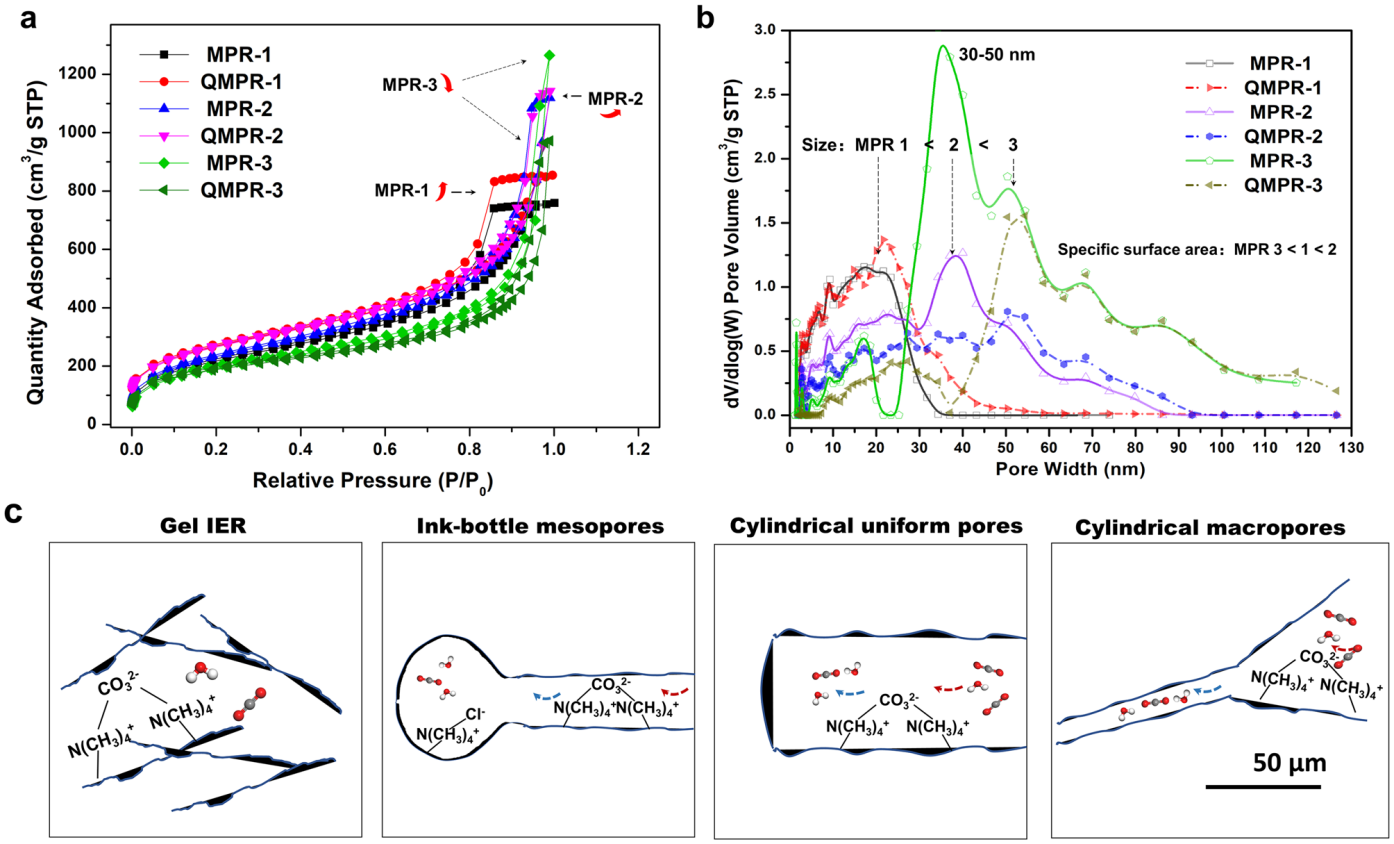
(**a**) N_2_ adsorption–desorption isotherms. (**b**) Pore size distributions calculated by density functional theory (DFT). (**c**) Pore characteristic schemes of gel IER, ink-bottle mesoporous, cylindrical uniform pores and cylindrical macropores.

N_2_ adsorption and desorption isotherms of different MPRs are shown in Fig. 2a. All MPRs exhibit type IV isotherms with hysteresis loops, which is associated with capillary condensation of nitrogen taking place in mesopores. The H2 hysteresis loop of MRP-1 indicates special ink-bottle-like mesopores with narrow opening, while H1 hysteresis loops of MRP-2 and MPR-3 generally suggesting cylindrical pores with more uniform size of resins^20^. As for MPR-3, it should have a certain amount of macropores (> 50 nm), as the isotherms showed no obvious adsorption saturation at large *P*/*P*_0_ where fits the zone of multilayer adsorption. Quaternization didn’t change the pore type of MPRs and QMPRs based on the same type of isotherms. However, the pore volumes of QMPR-2 and QMPR-3 drop significantly compared to MPRs, especially between the pore size of 30 and 50 nm (Fig. 2b). This could be primarily contributed by the swelling process, where it generally induce a 15–25% increase in bulk volume and squeeze mesopores of resins^21,22^. On the other hand, the swelling effect also result in higher surface area (Table 1) and higher amination efficiency, as the grafting of TMA may barely affect the pore structure of resin due to the small molecular size (0.66 nm) and monolayer grafting^23^. The decreasing surface area of QMPR-3 compare to MPR-3 may be due to the combined effect of swelling and amination.

**Table 1:** Summary of textural properties of resin samples. C and N: the weight content of carbon and nitrogen from element analysis, respectively. *S*_BET_ (m^2^ g^−1^): the specific surface area by BET method; *V*_DFT_ (cm^3^ g^−1^) and *D*_DFT_ (nm): the total pore volume and average pore size calculated by DFT method, respectively.

Resin samples	N (wt.%)	C/N	*S*_BET_ (m^2^ g^−1^)	*V*_DFT_ (cm^3^ g^−1^)	*D*_DFT_ (nm)
MPR-1	–	–	808	1.04	10.42
QMPR-1	1.30	55.95	975	1.24	11.51
MPR-2	–	–	864	0.92	21.64
QMPR-2	1.41	50.75	967	0.71	23.84
MPR-3	–	–	662	1.11	40.14
QMPR-3	1.39	51.97	636	0.62	46.68

### Quaternization with pore structure of MPRs

The CO_2_ capacity of QMPRs can be predicted by nitrogen content (*Q*_N_) or charge density (*Q*_c_) through Eq. (1) below^13,24^, which the results are listed in Table 2.

1 $$ {\text{CO}}_{2} + ({\text{N}}^{ + } )_{2} \cdot {\text{CO}}_{3}^{2 - } + {\text{H}}_{2} {\text{O}} = 2({\text{N}}^{ + } ) \cdot {\text{HCO}}_{3}^{ - } $$

**Table 2:** Capacity and efficiency of polymeric resins for adsorption. *ρ*_i_ (mmol g^−1^): ion charge density through Mohr titration; *Q*_N_ (mmol g^−1^): CO_2_ capacity calculated from N content; *Q*_i_ (mmol g^−1^): CO_2_ capacity calculated from *ρ*_i_; *Q*_400ppm_: experimental CO_2_ capacity at 400 ppm. FG: the grafted functional group (2[N(CH_3_)_4_]^+^·CO_3_^2−^) of QMPR.

Adsorbents	*ρ*_i_ (mmol g^−1^)	Capacity (mmol g^−1^)			FG efficiency (%)	
		*Q*_N_	*Q*_i_	*Q*_400ppm_	*η*_1_ (*Q*_*i*_/*Q*_N_)	*η*_2_ (*Q*_400ppm_/*Q*_i_)
QMPR-1	0.75	0.46	0.37	0.22	80.4	59.5
QMPR-2	0.93	0.50	0.46	0.28	92.0	60.9
QMPR-3	0.75	0.50	0.38	0.22	76.0	57.9

The ion exchange efficiency of functional group (FG), *η*_1_, is employed to measure the availability of QA group. Only the QA cations electrostatic interacting with carbonate ions are effective for CO_2_ adsorption. Gel type ion exchange resins (IER) generally have a high *η*_1_ of over 98%^25^. Compared with that, the relatively lower *η*_1_ of QMPR should be due to the uneven distribution of QA groups inside the mesopores. Ion exchange for carbonate requires two QA cations with proper distance. Cation distance larger or shorter than the size of one carbonate molecule between two adjacent cations will result in increased potential energy of ion exchange^26^. For gel type IER, as illustrated in Fig. 2c, the cations are randomly distributed in the three-dimensional network of crosslinked polymer. The strong repulsion between cations will result in a uniform cation distance of about 0.8 nm for IER with charge density of 3.4 mmol g^−1^, which provides proper space for counter anions with multivalence, e.g. carbonate, acetate, or phosphate anion^15,18^. During quaternization of QMPR, the diffusion of acyl group through micropores in gel type resins would have large resistance compared to diffusion through mesopores. The QA group would be concentrated inside the pores which can be indicated by the poor content of nitrogen, or large value of C/N. Uneven spatial distribution of cations, or cations with distance of far smaller or larger than the size of carbonate ion will result in poor ion exchange efficiency^26^. For QMPRs, the *η*_1_ should also be related to the pore shape. Compared to QMRP-1 with ink-bottle pores and QMPR-3 with macropores, QMPR-2 with cylindrical mesopores is expected to have QA cations with more uniform spatial distance. This should be the reason why QMPR-2 has relatively higher ion exchange efficiency.

### Mechanism of water effect on capacity of QMPRs

**Figure 3:**
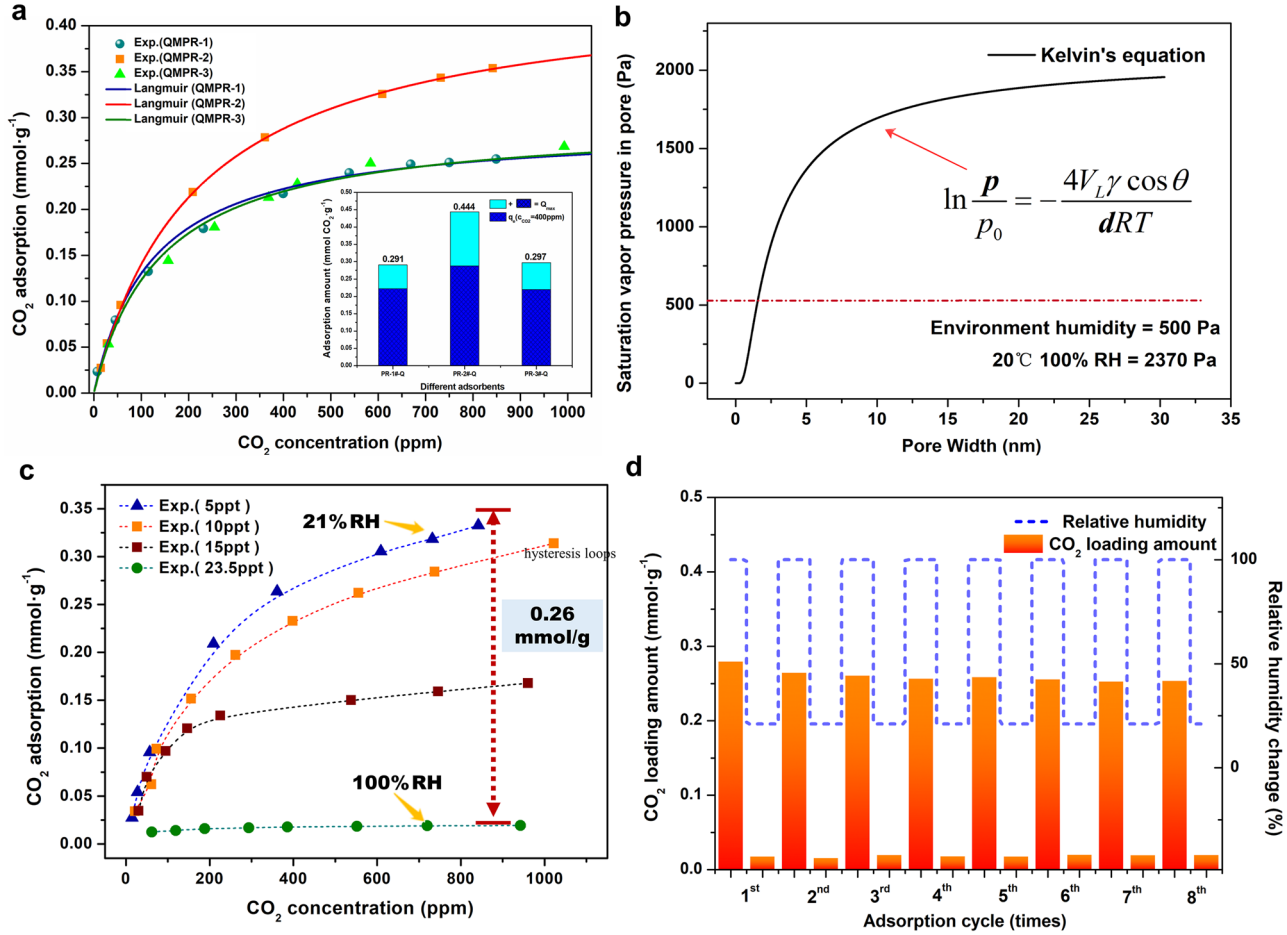
CO_2_ adsorption performance and cycling of QMPRs. (**a**) Adsorption isotherms at 20 °C and 21% RH. (**b**) Local relative humidity of pores caused by capillary condensation of water. (**c**) Adsorption isotherms of QMPR-2 at different RH. A stable swing capacity of 0.26 mmol g^−1^ can be obtained by adsorbing at 21% RH and desorbing at 100% RH. (**d**) Multiple cycles for CO_2_ adsorption by QMPR-2 at 400 ppm and desorbed at 100% RH.

CO_2_ adsorption isotherms of QMPR adsorbents are plotted in Fig. 3a, which exhibited Langmuir isotherm characteristics (Supplementary Fig. 3). No CO_2_ adsorption is detected in MPRs (Supplementary Fig. 4). QMPR-2 exhibits the largest CO_2_ capacity of 0.28 mmol g^−1^ under relative humidity (RH) of 21% and CO_2_ concentration of 400 ppm, while QMPR-1 and QMPR-3 have relatively lower CO_2_ capacity. This adsorption results consist with the ion exchange capacity of QMPRs. Table 2 shows that, for all the quaternized adsorbents, the functional group efficiencies at 400 ppm, *η*_2_, are close to 60%. Although *η*_2_ is larger than that of most solid amines^11,27^, it is still lower than that of gel type IER^13^. From thermodynamic point of view, the functional group efficiency under a certain CO_2_ partial pressure is determined by the equilibrium constant, which reflects the binding ability of adsorbents with CO_2_. For moisture swing adsorbents, the CO_2_ binding energy should be primarily affected by environmental humidity^24^ and hydrophilicity of material^17^. Due to the hydrophilic feature of QA groups^27^, QMPR has higher water sorption capacity compared to MPR (Supplementary Table 1). One can assume that the increased water content after amination is concentrated around the functional groups. Therefore, it is not surprising to find that QMPR and gel IER have similar hydrophilicity, or water content per functional group.

More influential effect goes to the capillary condensation of water taking place inside the pore channels, which changes the environmental humidity in local pores. The capillary condensation under equilibrium conditions is given by Kelvin’s equation:

2 $$ \ln \frac{p}{{p_{0} }} = - \frac{{2V_{L} \gamma \cos \theta }}{rRT} $$

*V*_*L*_ is the molar volume of water, *γ* is the surface tension of water, and *R* is the gas constant. For a hydrophilic surface (contact angle, *θ*, < 90°), the saturation vapor pressure inside the pores (*p*), as a function of pore width (*d*) and temperature (*T*), is smaller than that in bulk phase (*p*_*0*_). Condensation can, therefore, take place under higher relative humidity^28^. As illustrated in Fig. 3b, under water vapor partial pressure of 500 Pa at 20 °C (21% RH in ambient air), the local relative humidity in pores smaller than 10 nm could be over 30%. The increased number of water molecules surrounding by QA groups will increase the free energy of water dissociation for hydroxide ion and lead to decreased binding ability of adsorbents with CO_2_^24^. The moisture swing adsorption property of QMPR-2 is depicted by the CO_2_ adsorption isotherms at different relative humidity (Fig. 3c). By adsorbing 400 ppm CO_2_ at 21% RH, and desorbing at RH of 100%, a stable swing capacity of 0.26 mmol g^-1^ can be obtained (cyclic adsorption shown in Fig. 3d).

### Adsorption kinetics of QMPRs

**Figure 4:**
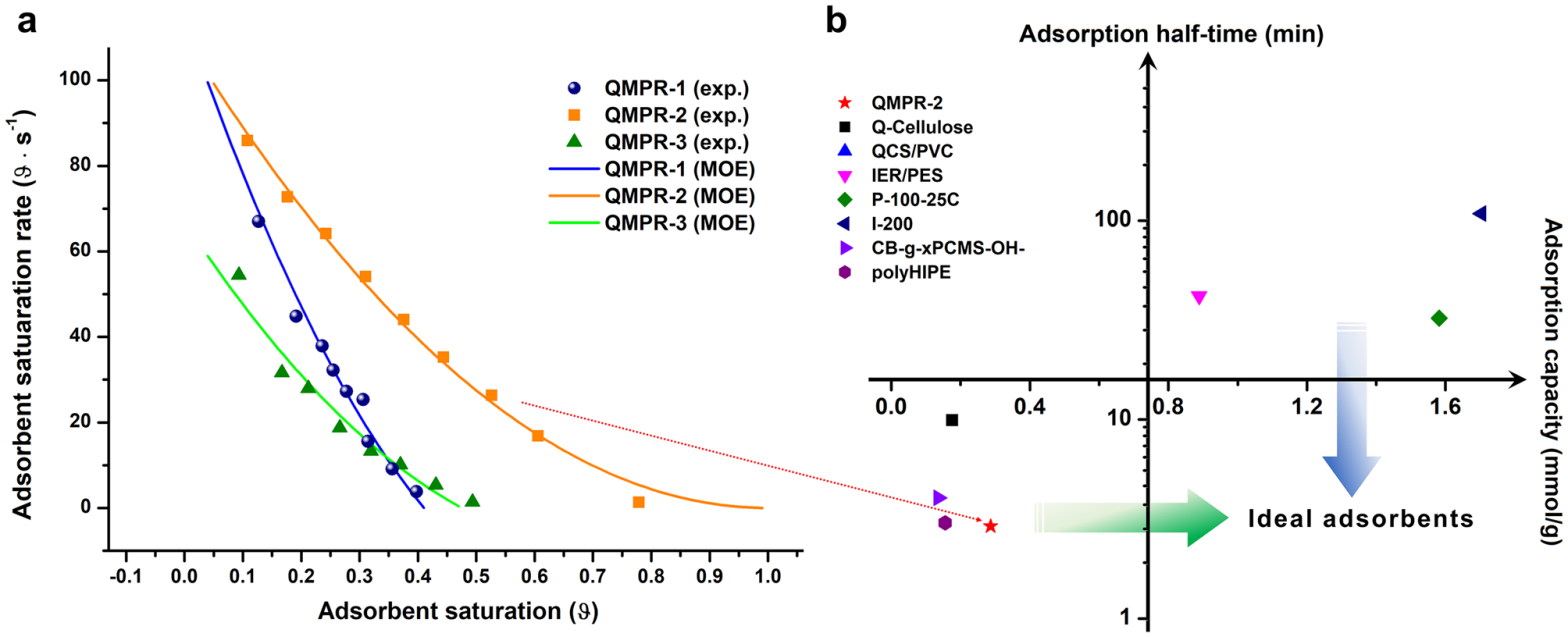
CO_2_ adsorption kinetics and comparison among MSA adsorbents. (**a**) Adsorption kinetics of QMPRs at 20 °C and 21% RH. (**b**) Adsorption quadrantal diagram of developed MSA adsorbents for DAC.

Figure 4a demonstrates that CO_2_ adsorption kinetics of QMPRs is governed by the surface area of adsorbent. QMRP-3, which owns the smallest surface area among adsorbents, has the lowest adsorption rate. QMPR-1 and QMPR-2 have similar surface area and their adsorption rates are also close at initial state. Meanwhile, it is interesting to find that the adsorption rate of QMPR-1 drops more rapidly with increased CO_2_ saturation. This should be due to the special ink-bottle-like micro-pore structure as indicated by the hysteresis loop of QMRP-1. Previous isothermal studies^24^ revealed that quaternary ammonium adsorbent will release part of hydrated water during CO_2_ adsorption, e.g. 2.7 to 3.7 mol H_2_O mol^−1^ CO_2_. During CO_2_ adsorption of QMPR-1, the released water could be gradually trapped by the ink-bottle-like microstructure. This would result in fast accumulation of humidity in local pores, and ultimately deteriorate its adsorption kinetics.

Adsorption half time of QMPRs, which is defined as the time to reach half of CO_2_ capacity, could be calculated through a mixed 1,2-order equation^29^ (Fitting result is shown in Supplementary Table 2). QMPR-2 exhibits an exciting feature of kinetics with half time of 2.9 min (Fig. 4b), which is the highest kinetics value reported among DAC adsorbents. Enhanced kinetics of MSA gives great opportunities to facilitate the deployment of direct air capture in large-scale carbon capture. Adsorption quadrantal diagram with two indicators of adsorption capacity and adsorption half-time is employed to evaluate the state-of-the-art MSA adsorbents for DAC. Due to the high extent of quaternization during sol–gel fabrication of commercial resin^24^, the MSA adsorbents based on commercial quaternized resins generally have CO_2_ capacity as high as 1.5 mmol g^−1^, and are scattered at quadrant I. Recently synthesized MSA adsorbents have high kinetics and located in quadrant III as they are bottom-up designed based on backbone with porous structure. The ideal MSA adsorbent, which has both high capacity and high kinetics, can be expected by grafting functional group into mesoporous resins during sol–gel fabrication process.

## Discussion

The moisture swing separation process, which adsorb CO_2_ at dry atmosphere and desorb CO_2_ at high relative humidity, provides a promising approach to low cost CO_2_ capture from air. In this work, the quaternary ammonium functional group was grafted onto mesoporous polymers to develop MSA adsorbent with high kinetics. In order to provide sufficient mass transfer channels for diffusion of functional group during grafting, Dichloromethane and methanol were selected as swelling agents as they can overcome steric hindrance and permeate reagents into active substitution sites at a sufficiently swelled state. The swelling effect also results in increased surface area and amination efficiency which are quite different from traditional amine functionalized adsorbents. Furthermore, cylindrical mesopores, which exhibits type IV isotherms with H1 hysteresis loops, are expected to have quaternary ammonium functional group with more uniform spatial distance and higher amination efficiency. The adsorbent synthesis employing proper swelling agents and polymer support with optimized mesoporous structure achieved adsorption half time of 2.9 min under atmospheric environment. This is the highest kinetics value reported among DAC adsorbents. Moreover, the behavior of water is significantly affected by mesopores, which further alters the binding energy of quaternary ammonium ion pair to CO_2_. Mesopores have lower saturation vapor pressure according to Kelvin’s equation capillary and have higher environmental humidity in local pores. The increased hydration water can distinctly decrease the binding energy of quaternary ammonium ion pairs to CO_2_ through the Brønsted base mechanism. This can result in poor functional group efficiencies at 400 ppm, or moisture swing ability, compared to gel type ion exchange resin. Further improvement of MSA adsorbent with high capacity and kinetics can be expected by grafting quaternary ammonium into mesoporous matrix with hydrophobic groups.

## Methods

### Fabrication of QMPRs

Commercial nonpolar porous resins (XAD-4, XAD-16, and XAD-1180N, purchased from Aladdin, China) were pretreated by 0.1 M HCl, 5 wt.% NaOH and deionized (DI) water to remove the impurities. After dried at 60 °C under vacuum, 4.00 g mesoporous resin (MPR) sample was placed in a four-necked flask (equipped with constant pressure funnel, reflux condenser, thermometer, and mechanical stirring). 20 mL dichloromethane (CH_2_Cl_2_) was added as solvent and the mixture was stirred for 12 h to fully swell the microspheres. Chloroacetyl chloride (reagent) and powdered anhydrous aluminum chloride (Lewis catalyst) were slowly added to the mixture under N_2_ protection (molar ratio of MPR to reagent to catalyst is 1:1:1). After 4.5 h room-temperature reaction, the mixture was suction-filtered and washed with tetrahydrofuran, 0.1 M HCl, and DI water in sequence, until chlorine ion could not be detected in the solution. The resulting light-brown microspheres were named as MPR-CA-1, MPR-CA-2, and MPR-CA-3, respectively.

The 4.00 g MPR-CA then swelled in 20 mL MeOH for 6 h at room temperature. Accompanied with 60 wt.% trimethylamine (TMA, 3.0 *e.q.* based on chloroacetyl chloride), the mixture was reacted in the flask with an oil bath (fitted reflux) pre-heated to 70 °C. After 4 h, the obtained resin microspheres (MPR-Cl) were packed in a column, washing with MeOH and DI water to remove the residuals. The obtained product was dried under vacuum at 60 °C for 12 h. After ion-exchange with 1 M Na_2_CO_3_ solution, the synthesized quaternary ammonium anion exchange mesoporous resins (QMPRs) were produced.

### Characterization

Pore characteristics of MPRs and QMPRs were analyzed using N_2_ adsorption/desorption isotherms (ASAP2020, Micromeritics, USA). Total pore volume was based on the adsorbed amount of N_2_ at *P*/*P*_0_ = 0.99. The specific surface area was calculated using Brunauer–Emmett–Teller (BET) method (0.01 < *P*/*P*_0_ < 0.1), and pore-size distribution was obtained by density functional theory (DFT). Chemical structure of resin samples was identified by a Fourier transform infrared spectrometer (FTIR, Digilab BioRad FTS 6000 spectrometer, ATR mode), which scans from 4000 to 400 cm^−1^. The element contents, including carbon, hydrogen, and nitrogen of resins, were determined though vario MAX cube and Elementar equipment. Surface morphology of resin samples were observed by a Hitachi SU-8010 scanning electron microscope (SEM) with an accelerating voltage of 20 kV. The Mohr titration was used to quantify the chloride ion amount in exchanging residue of QMPRs. By getting charge density (*Q*_c_) of QMPR, an ideal CO_2_ capacity can be calculated from it. Thus, it further gives the ion exchange efficiencies (*η*_1_) of functional group (FG) and FG efficiencies at 400 ppm (*η*_2_) of QMPRs.

### CO2 adsorption measurements

CO_2_ adsorption isotherms and kinetics were performed in a self-made system (Supplementary Fig. 5). CO_2_ leakage was tested before each measurement. By repeatedly injecting 0.5 ml of CO_2_ using syringe, the leakage rate was tested as 1.4 × 10^–4^ ppm s^−1^ under a CO_2_ concentration difference of 500 ppm. This verifies that CO_2_ leaking issue could be negligible compared to the injected amount.

### Isothermal and kinetics models

4.00 g QMPR was loaded in the reaction chamber, dried by ultra-high-purity (UHP) N_2_ before adsorption. Relative humidity (RH) was controlled at 21% under 20 °C. CO_2_ was injected into chamber repeatedly once adsorption equilibrium was reached. An isotherm related between CO_2_ concentration and adsorbed amount can be obtained as:

3 $$ Q_{{\text{e}}} = \frac{{Q_{inj} - c_{e} V_{s} }}{{m_{ad} V_{m} }} $$

where *Q*_e_ and *Q*_inj_ are CO_2_ adsorbed and injected volume. *c*_e_ is the CO_2_ concentration at equilibrium. *V*_s_ is the volume of the system (8 L). *m*_ad_ is the adsorbent weight. *V*_m_ is the molar volume.

Langmuir, Freundlich, and Temkin isotherm models are employed to fit the MSA equilibrium. These models can be reformed by linear relationship between monotonic function of *Q*_*e*_ and *P*.

4 $$ {\text{Langmuir isotherm:}}\quad \frac{1}{{Q_{e} }} = \frac{1}{{Q_{\max } }} + \frac{1}{{K^{\prime}Q_{\max } P}} $$

5 $$ {\text{Freundlich isotherm:}}\quad \ln Q_{e} = A_{1} + B_{1} \ln P $$

6 $$ {\text{Temkin isotherm:}}\quad Q_{e} = A_{2} + B_{2} \ln P $$

where *Q*_e_ and *Q*_max_ are CO_2_ adsorbed amount at equilibrium and 100% saturation, respectively. *A*_*1*_, *B*_*1*_, *A*_*2*_, and *B*_*2*_ are adsorption parameters calculated by linear fitting. *K’* is the effective equilibrium constant of adsorption.

The normalized adsorption rate at a certain CO_2_ concentration is determined by calculating the slope of *θ*_*t*_ ~ *t* curve. By measuring the CO_2_ concentration (*C*_*t*_) at time *t*, the saturation of adsorbent (*θ*_*t*_) can be calculated as:

7 $$ \theta (t) = \frac{{Q_{inj} - C_{t} V_{s} }}{{Q_{\max } }} $$

Pseudo-first-order (PFO), pseudo-second order (PSO), and mixed 1,2-order (MOE) rate models are employed to investigate the adsorption kinetics, which expressed as:

8 $$ {\text{PFO rate model:}}\quad Q_{t} = Q_{{e_{1} }} (1 - e^{{ - k_{1} t}} ) $$

9 $$ {\text{PSO rate model:}}\quad Q_{t} = \frac{{k_{2} {Q_{{e_{2} }}}^{2} t}}{{1 + k_{2} {Q_{{e_{2} }}}^{2} t}} $$

10 $$ {\text{MOE rate model}}:\quad Q_{t} = Q_{{e_{1} }} (1 - e^{{ - k_{1} t}} ) + \frac{{k_{2} {Q_{{e_{2} }}}^{2} t}}{{1 + k_{2} {Q_{{e_{2} }}}^{2} t}} $$

11 $$ f_{2} = \frac{{k_{2} Q_{{e_{2} }} }}{{k_{1} + k_{2} Q_{{e_{2} }} }} $$

where *Q*_*t*_ (mmol g^−1^) is CO_2_ adsorbed amount at time *t*, *k*_*1*_ and *k*_*2*_ are rate constants for PFO and PSO models, respectively. *f*_*2*_ is the percentage of PSO equation in MOE rate model.

### H_2_O adsorption analysis

H_2_O adsorption capacity of MPRs and QMPRs were obtained using a gravimetric method. Sample of resin (~ 1.00 g) were first dried in the chamber using UHP N_2_, note *m*_*d*_ (g) as its dry mass. Humid N_2_ (N_2_ pass through humidity controller) was then introduced to the chamber, and record *m*_*w*_ (g) as the mass of sample saturated at a certain RH. This process was operated multiple times until *m*_*w*_ obtained from the electronic balance was stable. H_2_O adsorbed amount of each sample, *Q*_*w*_ (mmol g^−1^), was expressed as:

12 $$ Q_{w} = \frac{{1000(m_{w} - m_{d} )}}{{18m_{d} }} $$

## Supplementary Information

Supplementary Information.
