# Supplementary material for: Quaternary functionalized mesoporous adsorbents for ultra-high kinetics of CO2 capture from air
Source: Sci Rep. 2020 Dec 8;10:21429. doi: 10.1038/s41598-020-77477-1 (PMC7722900; doi:10.1038/s41598-020-77477-1)
Supplement: Supplementary file 1 — Supplementary Information. [file 41598_2020_77477_MOESM1_ESM.doc]

**Supplementary Information for**

**Quaternary Functionalized Mesoporous Adsorbents for Ultra-high Kinetics of CO_2_ Capture from Air**

# Tao Wang^1, *^, Xinru Wang^1^, Chenglong Hou^1^, Jun Liu^2^

^1^State Key Laboratory of Clean Energy Utilization, College of Energy Engineering, Zhejiang University, Hangzhou 310027, P. R. China

^2^School of Electric Power, North China University of Water Resources and Electric Power, Zhengzhou 450045, China

^*^Corresponding author: [oatgnaw@zju.edu.cn](mailto:oatgnaw@zju.edu.cn)


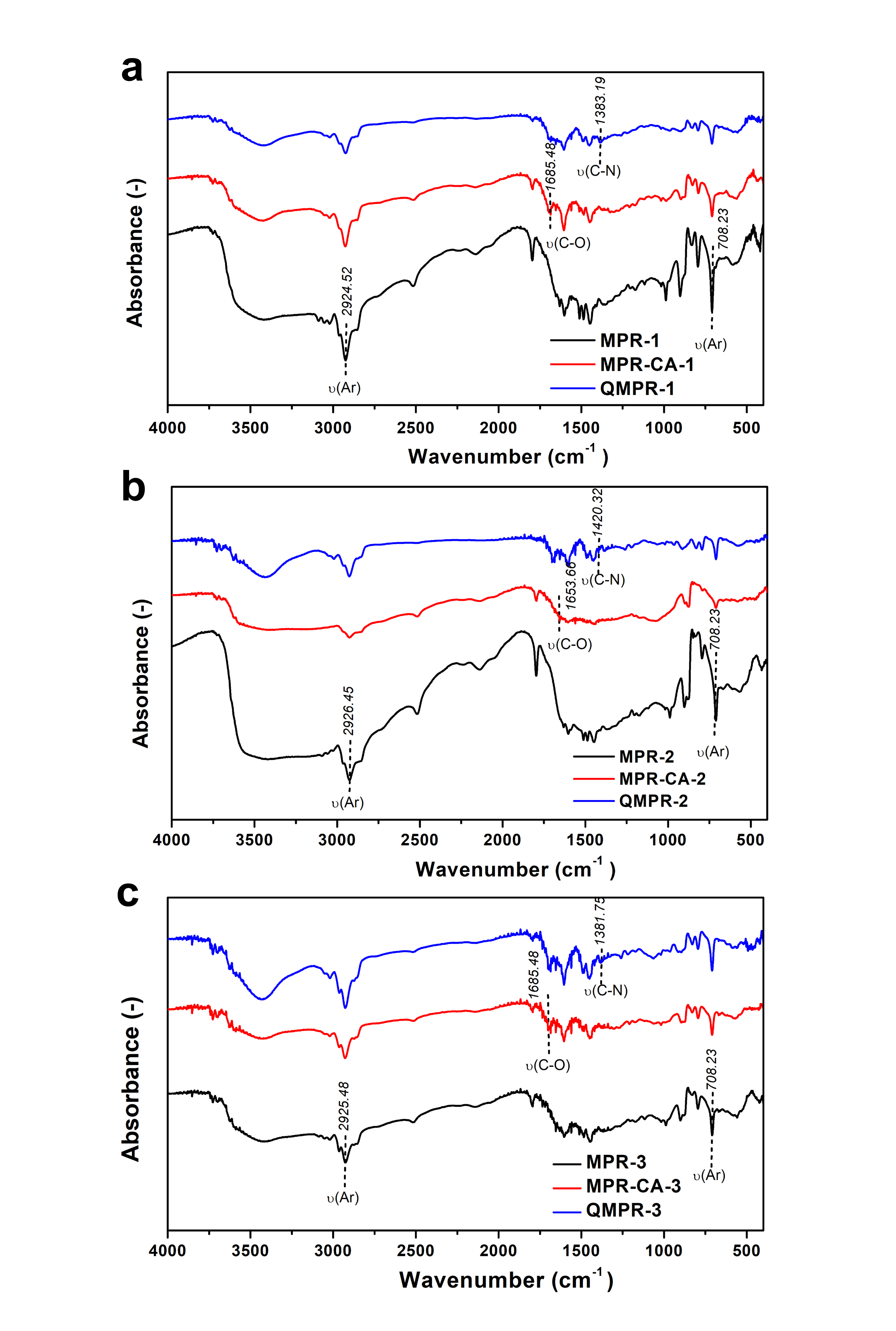


**Supplementary Figure 1. FTIR spectrum of MPRs, MPR-CAs and QMPRs.** **(a)**, **(b)** and **(c)** represent spectrum variations for three resin species -1, -2 and -3, respectively. The high adsorption peaks observed at 2925 cm^-1^ and 709 cm^-1^ of MPRs show characteristic vibration of polystyrene skeletons. After Step I, the intensity of characteristic peak at 709 cm^-1^ reduced, while a new stretching vibration at 1685 cm^-1^ appears. This new peak could correspond to the change of functional groups from styryl to acyl. For QMPRs, intensity of C−N stretching at 1380 cm^−1^ increases and vibration at 1022 cm^−1^ widen. This indicates that quaternary ammonium functional groups were successfully grafted onto MPRs (Step II and III).


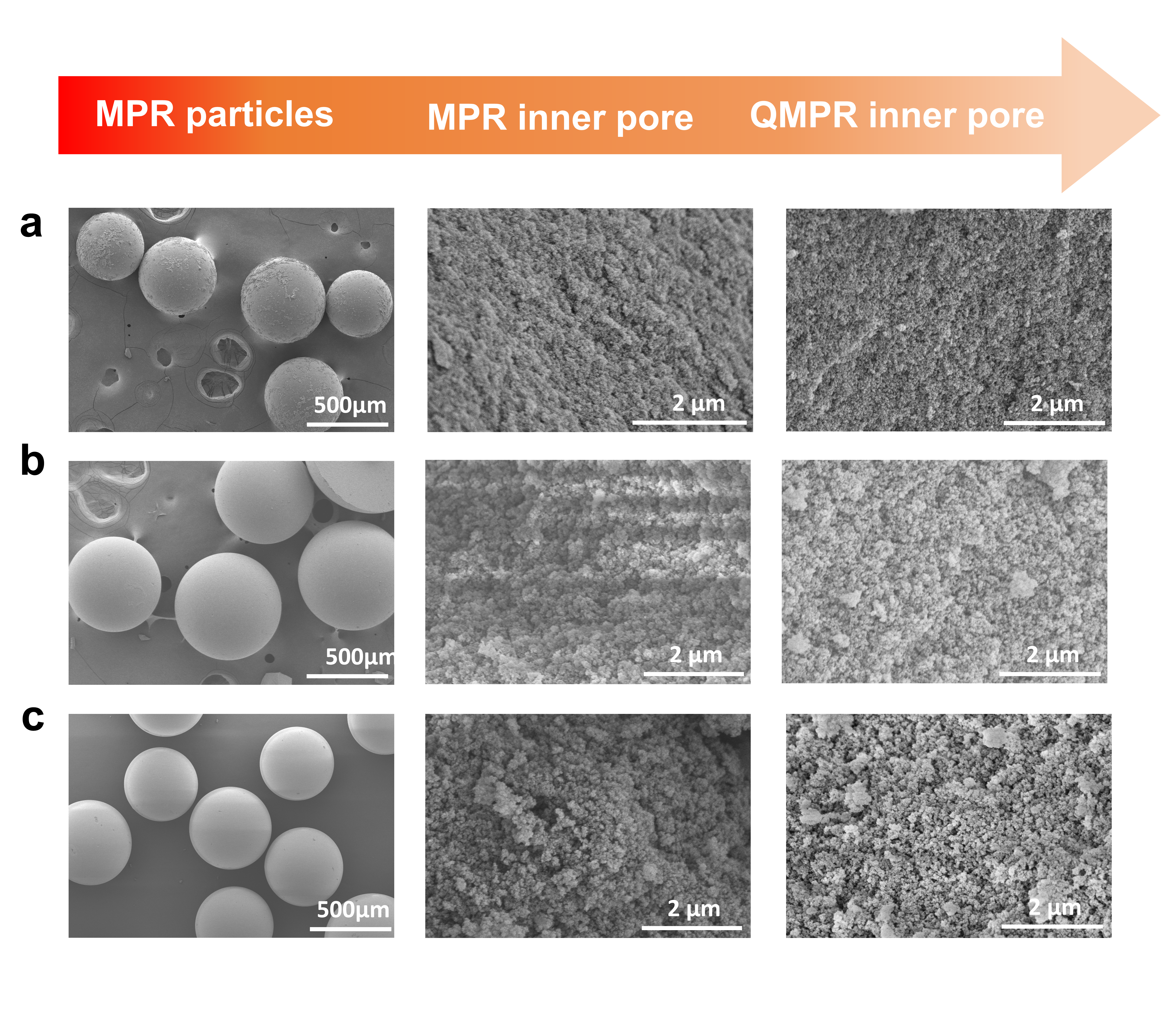


**Supplementary Figure 2. SEM images of adsorbent particles.** **(a)**, **(b)** and **(c)** represent MPR-1, -2 and -3. From left to right represent MPR particles at scale bar of 500 μm, its inner pore morphology at scale bar of 2 μm, and QMPR inner pore morphology at scale bar of 2 μm. MPRs demonstrate that the particle sizes are similar (500-600 μm).


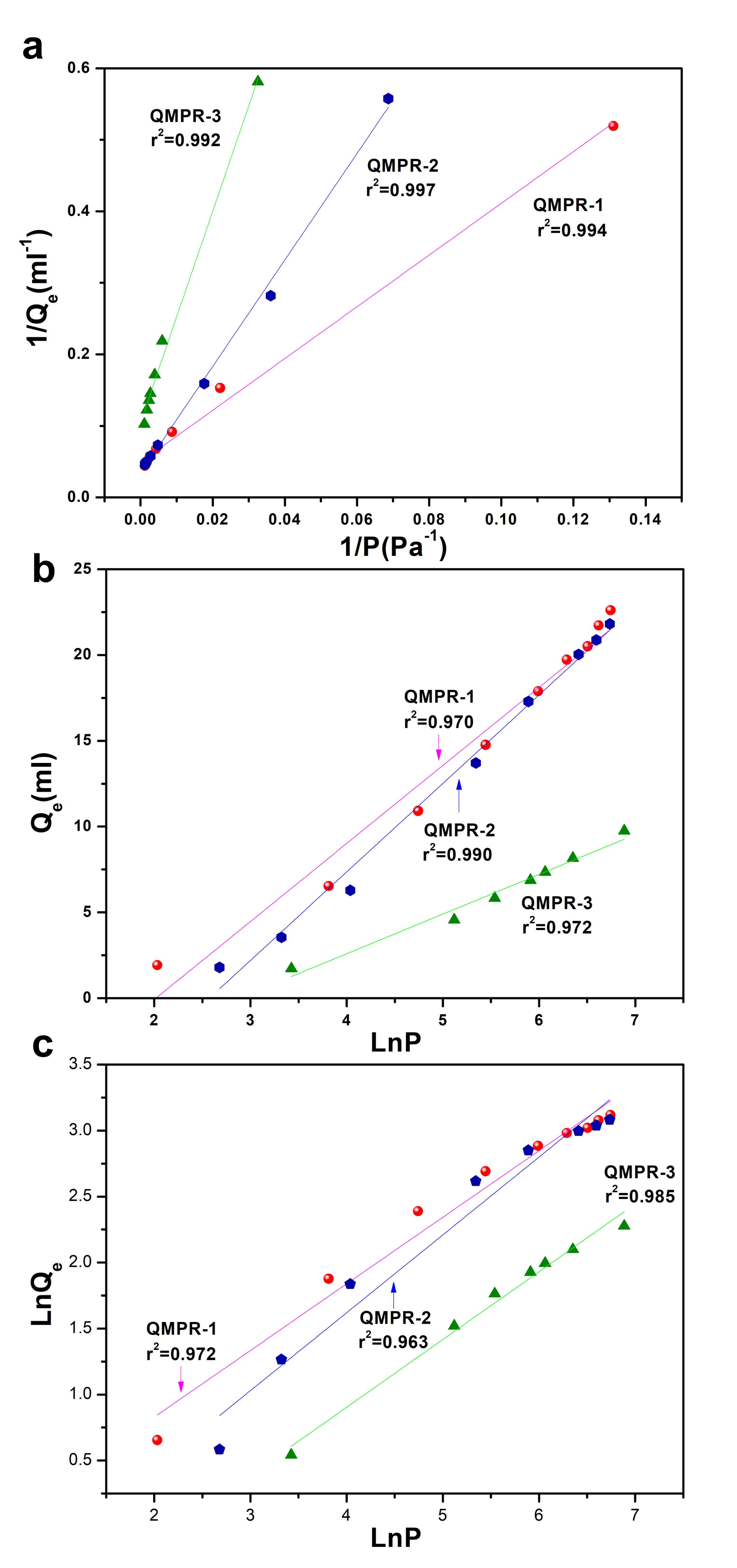


**Supplementary** **Figure 3.** **Linear fitting of CO_2_ adsorption isotherm models.** **(a)** Langmuir model. **(b)** Freundlich model. **(c)** Temkin model. r is fitting standard deviation. Each one contains comparison between QMPRs.


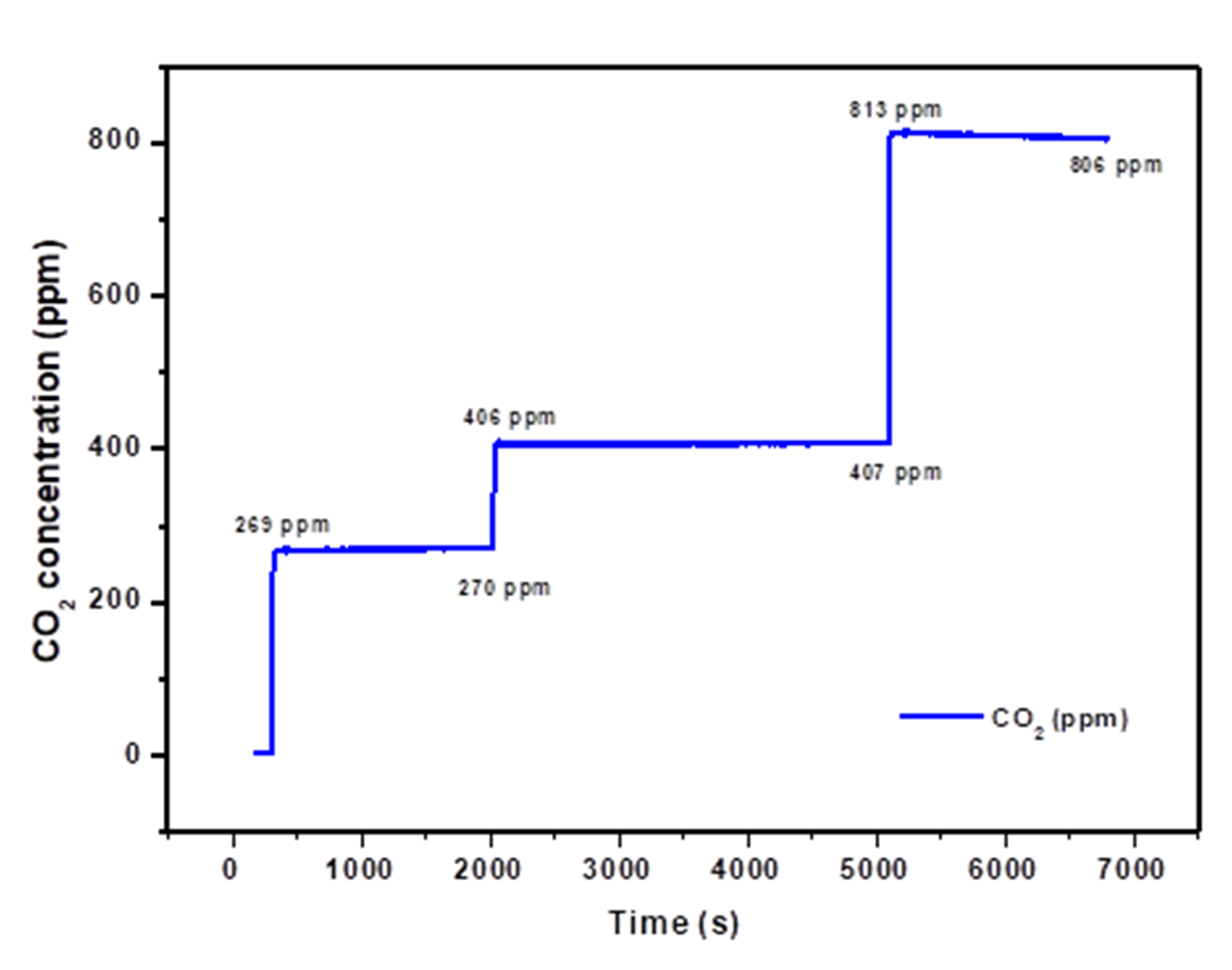


**Supplementary Figure 4. CO_2_ adsorption equilibrium of MPR**. Giving the pretesting adsorption performance of MPR-2, CO_2_ concentrations could stable at increasing levels by injecting more CO_2_, it confirmed that no physical adsorption contributed to the adsorption process.


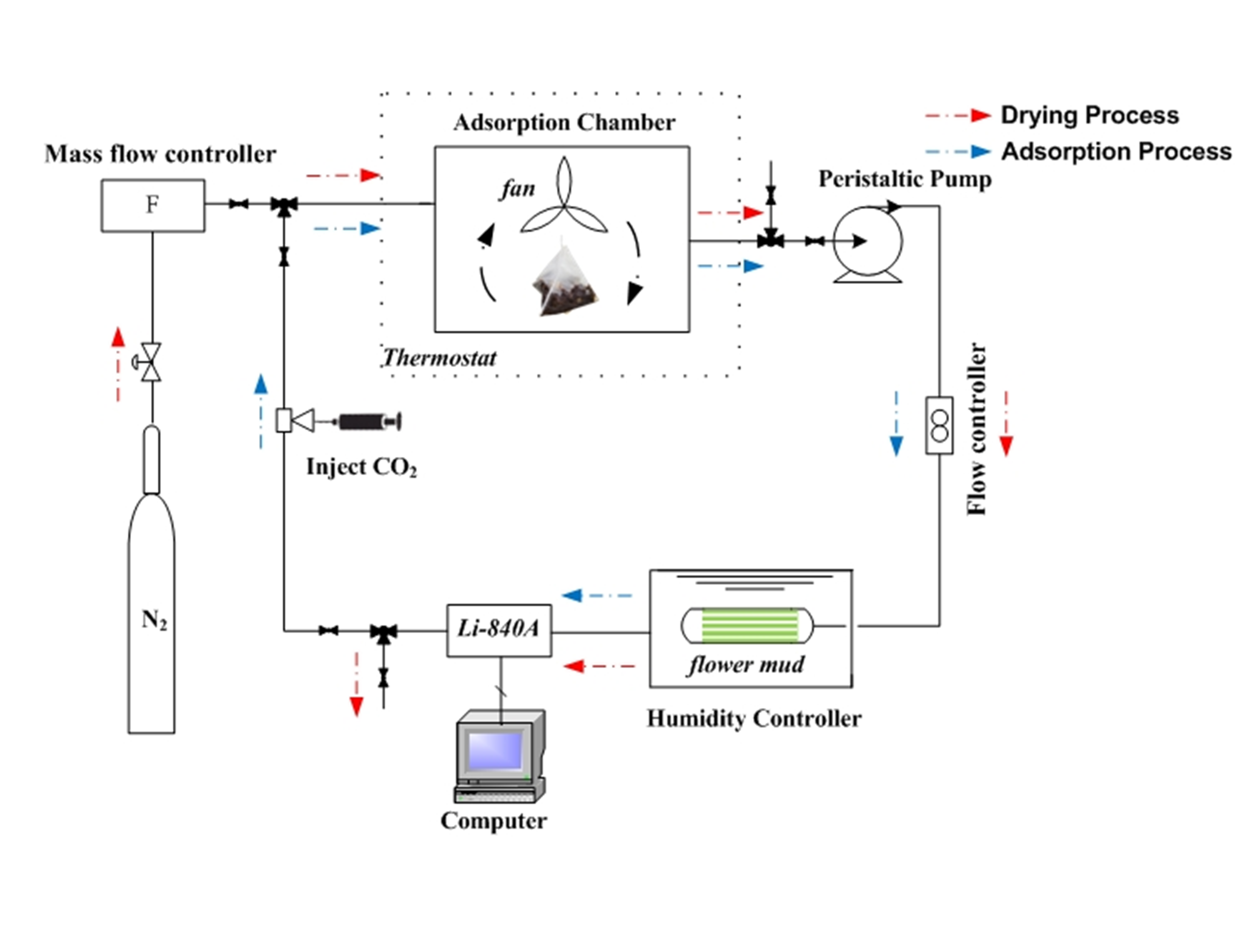


**Supplementary Figure 5. Experimental set-up for CO_2_ adsorption measurements.** The system mainly contains a reaction chamber (8.0 L, with a thermostat to control temperature), an infrared gas analyzer (LI-840A, LI-COR, dynamic monitoring CO_2_ and vapor concentration), and an inner environment humidity controller.

| **Resin samples** | **Adsorption amount**  **(mmol g^-1^)** | **Adsorption amount**  **(mmol mol^-1^ FG)** |
| --- | --- | --- |
| MPR-1 | 6.87 | - |
| QMPR-1 | 13.14 | 27.19 |
| MPR-2 | 7.15 | - |
| QMPR-2 | 14.22 | 21.44 |
| MPR-3 | 7.13 | - |
| QMPR-3 | 13.97 | 28.22 |
| I200^1^ | ca.27 | 20.95 |

**Supplementary Table 1.** Water Adsorption Properties of MPRs and QMPRs. FG indicates grafted functional group ([2N(CH_3_)^4+^]·CO_3_^2-^) of QMPR.

| **Resin samples** | **k_1_** | **k_2_** | **f_2_** | **r^2^** |
| --- | --- | --- | --- | --- |
| QMPR-1 | 0.611 | 4.225 | 2.437 | 0.996 |
| QMPR-2 | 0.013 | 1.157 | 0.974 | 0.998 |
| QMPR-3 | 0.281 | 1.818 | 2.103 | 0.981 |

**Supplementary Table 2. Fitting Results of the Kinetic Parameters.** *k_1_* and *k_2_* are rate constants for PFO and PSO models. *f_2_* is the percentage of PSO equation in MOE rate model.

| **Adsorbent** | **Temperature (°C)** | **Adsorption capacity**  **(mmol g^-1^)** | **Adsorption half-time (min)** | **Reference** |
| --- | --- | --- | --- | --- |
| QMPR-2 | 20 | 0.19^1^ | 2.9 |  |
| Q-Cellulose | 25 | 0.18^1^ | 9.8 | 2 |
| QCS/PVC | 20 | 0.181^1^ | 10.7 | 3 |
| IER/PES | 20 | 0.93^1^ | 52.1 | 4 |
| P-100-25C | RT | 1.58^1^ | 31.8 | 5 |
| I-200 | 24 | 1.70^1^ | 106 | 5 |
| CB-g-xPCMS-OH- | RT | 0.14^2^ | 1.1^3^ | 6 |
| polyHIPE | RT | 0.49^2^ | 3.9^3^ | 7 |

**Supplementary Table 3.** **Comparison of Adsorption Half-Time for Different Quaternary Ammonium-Based Sorbents.** ^1^The maximal CO_2_ adsorption capacity at 400 ppm of CO_2_. ^2^The moisture-swing size of the sorbents. ^3^The adsorption half-time was obtained with the Engauge Digitizer 4.1 program.

**REFERENCE**

1. Wang, T., Lackner, K. S. & Wright, A. B. Moisture-swing sorption for carbon dioxide capture from ambient air: A thermodynamic analysis. *Phys. Chem. Chem. Phys.* **15**, 504–514 (2013).

2. Hou, C., Wu, Y., Wang, T., Wang, X. & Gao, X. Preparation of Quaternized Bamboo Cellulose and Its Implication in Direct Air Capture of CO 2. *Energy and Fuels* **33**, 1745–1752 (2019).

3. Song, J. *et al.* Quaternized Chitosan/PVA Aerogels for Reversible CO2 Capture from Ambient Air. *Ind. Eng. Chem. Res.* **57**, 4941–4948 (2018).

4. Wang, T., Liu, J., Huang, H., Fang, M. & Luo, Z. Preparation and kinetics of a heterogeneous sorbent for CO2 capture from the atmosphere. *Chem. Eng. J.* **284**, 679–686 (2016).

5. Shi, X., Li, Q., Wang, T. & Lackner, K. S. Kinetic analysis of an anion exchange absorbent for CO2 capture from ambient air. *PLoS One* **12**, 1–12 (2017).

6. He, H. *et al.* Carbon black functionalized with hyperbranched polymers: Synthesis, characterization, and application in reversible CO2 capture. *J. Mater. Chem. A* **1**, 6810–6821 (2013).

7. He, H. *et al.* Porous polymers prepared via high internal phase emulsion polymerization for reversible CO2 capture. *Polymer (Guildf).* **55**, 385–394 (2014).
